# Acute kidney injury increases the rate of major morbidities in cytoreductive surgery and HIPEC^☆^

**DOI:** 10.1016/j.amsu.2018.09.036

**Published:** 2018-09-26

**Authors:** Samer A. Naffouje, Kiara A. Tulla, Regina Chorley, Nancy Armstrong, George I. Salti

**Affiliations:** a Department of General Surgery, University of Illinois Hospital and Health System, Chicago, IL, USA; b Division of Surgical Oncology, Edward Hospital Cancer Center, Naperville, IL, USA; c Division of Surgical Oncology, University of Illinois at Chicago Hospital and Health System, Chicago, IL, USA

**Keywords:** CRS and HIPEC, AKI, Major morbidity

## Abstract

**Introduction:**

Acute kidney injury (AKI) following cardiovascular surgery has been shown to increase costs and overall morbidity and mortality. The incidence, risk factors, and outcomes of AKI following other types of major surgeries have not been as well characterized. We sought to study the incidence of AKI following cytoreductive surgery (CRS) and hyperthermic intraperitoneal chemotherapy (HIPEC) per the Kidney Disease: Improving Global Outcomes (KDIGO) criteria.

**Materials and methods:**

Patients undergoing CRS and HIPEC between 2013 and 2015 were included. Demographic and perioperative data were compared between patients who experienced AKI versus controls using appropriate statistical analysis between categorical and continuous variables. AKI was recorded by a Certified Professional in Healthcare Quality (CPHQ) and defined as a rise in serum creatinine by ≥ 0.3 mg/dL within 48 h (KDIGO criteria).

**Results:**

Fifty-eight consecutive patients undergoing CRS and HIPEC were included. Twelve (20.7%) patients were recorded to develop AKI. This was the most common complication recorded by the CPHQ member. There was one 30-day mortality secondary to cerebral infarction. AKI patients had a longer hospitalization period (14.2 ± 6.9 vs. 9.5 ± 3.3 days, p = 0.002), and a higher rate of major complications (50.00% vs. 15.21%; p = 0.018). Readmission rate was similar (p = 0.626). Multivariate regression identified excessive blood loss during surgery as a major predictor of AKI occurrence, and pre-existing comorbidities and postoperative AKI as predictors of major morbidities following CRS and HIPEC.

**Conclusion:**

AKI following CRS and HIPEC appears to be a common complication which is associated with further major morbidities. Current quality improvement programs may be under-reporting this incidence.

## Highlights

• We aim to study the incidence of acute kidney injury and renal recovery following cytoreductive surgery (CRS) plus hyperthermic intraperitoneal chemotherapy (HIPEC) per the Kidney Disease Improving Global Outcomes (KDIGO) criteria.
• Fifty-eight patients who underwent CRS and HIPEC at our institution were included over 2 years.
• AKI was the most common complication leading to a longer hospitalization and a higher rate of other major complications.
• The use of Mitomycin C as the HIPEC agent, as well as longer surgeries with increased blood loss were the only predictors of AKI occurrence. Our intra- and postoperative fluid management was not different between the AKI and non-AKI group.

## 1 Introduction

Acute kidney injury (AKI) has been increasingly recognized as a postoperative complication with potentially serious consequences [1,2]. About 30–40% of surgical patients suffer from AKI in the postoperative setting which is significantly higher than the 5–7.5% seen in all acute care hospitalizations [3]. Moreover, AKI accounts for up to 20% of admissions into intensive care units (ICU) [1,4]. A large proportion of our knowledge of the surgery-related AKI is derived from the cardiovascular literature [3,5] where the occurrence of AKI is estimated to reach 30% and has been associated with increased morbidity and mortality [6,7], higher costs [2], and worse survival [8].

To date, only a handful of studies evaluated the incidence of AKI in patients undergoing cytoreductive surgery with hyperthermic intraperitoneal therapy (CRS and HIPEC) [[9], [10], [11]]. However, kidney injury related to cisplatin, a known nephrotoxic agent, was a major contributor to the occurrence of AKI. Nonetheless, the specific impact of AKI on the surgical outcomes was not assessed.

The lack of uniform measures and varying definitions of AKI has resulted in a wide discrepancy in reporting its postoperative incidence which ranges from 1 to 31% [12]. Since 2004, consensus criteria were developed to define AKI. To provide consistency at our institution, AKI is recorded on all inpatient admissions by a Certified Professional in Healthcare Quality (CPHQ) trained to capture the appropriate data. Whether AKI, as a sequela of CRS and HIPEC rather than an adverse event of the intraoperative chemotherapeutic agent, worsens the short- and long-term outcomes remains unanswered, mainly due to the lack of well-established risk factors for AKI in the context of CRS and HIPEC surgery.

The scarcity of reports and the opportunity for improved standardization resulting from the international definition of AKI endorsed by Kidney Disease Improving Global Outcomes (KDIGO) in 2012 inspired us to evaluate our incidence of AKI in patients undergoing CRS and HIPEC at our community hospital as one of the complications associated with the procedure. With the aim of evaluating this group of individuals and understanding more thoroughly the risk factors associated with this perioperative morbidity in this patient population.

## 2 Methods

### 2.1 Study population

All patients prospectively identified with a variety of primary malignancies with peritoneal carcinomatosis or sarcomatosis that necessitated CRS and HIPEC between June 2013 and July 2015 at our institution were evaluated for inclusion in our study. Patients were excluded from this case cohort if they were identified to have chronic kidney disease as defined by our CPHQ. Our criteria for this diagnosis are established by a constant decrease in the glomerular filtration rate (GFR <60 ml/min per 1.73 m^2^) for >3 months, irrespective of cause, and was classified into five stages based on the level of GFR according to the KDIGO definition [13]. These data were prospectively maintained under an approved Institutional Review Board (IRB) protocol and listed in the National Clinical Trial registry (NCT02082886).

Patient-specific comorbidities that may hinder renal function in the perioperative period were accounted for such as the established diagnoses of hypertension, diabetes mellitus, cardiac disease, or systemic autoimmune diseases such as systemic lupus erythematous. The presence of any of these conditions would reflect in the patient receiving a Charlson comorbidity score of ≥1 as a metric to quantitatively measure the impact of existing diseases on the outcome. The Charlson index assesses patients’ risk of 10-year mortality based on existing controlled or uncontrolled chronic illnesses [14,15]. We used the Clavien-Dindo classification system to grade the postoperative complications. Grades I (any deviation from the normal postoperative course without the need for surgical, endoscopic, and radiological interventions, or within the allowed pharmacological treatments) and II (requiring pharmacological treatment with drugs other than such allowed for grade I, blood transfusion, or parenteral nutrition) were regarded as minor complications, whereas grades III (requiring a surgical, endoscopic, or radiological intervention), IV (a life-threatening complication requiring critical care management), and V (mortality) were considered to be major events [16].

### 2.2 Surgical technique

Our treatment consisted of tumor resection and removal of the involved organs and peritoneum, as deemed safe [17]. HIPEC was performed using the closed abdomen technique at 42–43 °C. Intraperitoneal chemotherapy regimens used included Mitomycin C (40 mg); Cisplatin (45 mg/L) ± Doxorubicin (15 mg/L); or, Melphalan (50 mg/m2). Peritoneal Carcinomatosis Index (PCI) was used to score the extent of peritoneal involvement at the time of surgery as reported in the 13-region and lesion size system [18]. Completeness of cytoreduction score (CC) was reported as CC0 for no residual disease, CC1 for microscopic residual disease (<0.25 cm), CC2 for macroscopic residual disease (0.25–2.5 cm), and CC3 for gross residual disease (>2.5 cm).

Our fluid management protocol is applied to maintain end-organ perfusion. Intra-operative fluid management included a continuous baseline infusion of crystalloids, aiming at a urinary output of at least 0.5 cc/kg/hr. If necessary, norepinephrine was started to keep mean arterial blood pressure at ± 20% of baseline values per the anesthesiologist. Arterial blood gas analyses were checked as needed to monitor signs of tissue hypoperfusion and volume trials were initiated if defined urinary output was not achieved and/or signs of impaired microcirculation were present. Post-operative fluid management was goal-directed at urine output of at least 0.5 cc/kg/hr.

### 2.3 AKI diagnostic criteria

Kidney Disease Improving Global Outcomes (KDIGO) criteria for AKI diagnosis are used at our institution per our CPHQ member. Occurrence of AKI was defined as a rise is serum creatinine (sCr) of ≥0.3 mg/dL within 48 h of surgery. Laboratory tests, including hematology and basic chemistry panels, were drawn immediately postoperatively and on daily basis until the day of discharge. Since our aim is to examine the immediate impact of AKI on the surgical morbidity, our follow up period was limited to the main admission during which the patients received the CRS and HIPEC.

### 2.4 Statistical analysis

In this study, the primary endpoint was to describe the AKI incidence in a single institution's cohort of CRS and HIPEC, and its correlation with the occurrence of other major morbidities. Our secondary endpoint was the identification of perioperative factors that would predict the occurrence of AKI. Continuous variables are presented as the means ± standard deviations and were compared using Student's *t*-test, or the non-parametric Mann–Whitney test for 2 groups or using the ANOVA for >2 groups. Categorical variables are presented as numbers (percentages) and were compared across groups using the χ^2^ or Fisher's exact test, as appropriate. All statistical analyses were conducted by IBM SPSS software version 23 for Windows (Armonk, NY: IBM Corp). P values ≤ 0.05 were considered significant statistically. Renal recovery was classified as complete when sCr returned to a level less than 50% above baseline at discharge.

Regarding the power of the study, the initial prediction was that 50–60 patients would be needed to achieve >80% power if a predicted 25% of the patients would demonstrate >0.3 mg/dL change in their baseline creatinine within 48 h of surgery compared to the non-AKI patients. The estimated percentage of AKI occurrence was derived from the published literature addressing AKI occurrence following major surgical intervention.

## 3 Results

The initial fifty-eight consecutive patients who underwent CRS and HIPEC and met the inclusion criteria between June 2013 and July 2015 at Edward Hospital were included in this study. After collecting data on 58 patients between June 2013 and July 2015, we documented that 20% of the sample's (12 patients) creatinine drifted into the AKI range (≥0.3 mg/dL within 48 h of surgery). The change in the creatinine between the baseline and postop values (within 48 h postoperatively) in the AKI group was 0.52 ± 0.46 compared to the non-AKI group which was 0.13 ± 0.15 (N = 46). The calculated power of our study as reported in the fifty-eight patients was 82.6%, thus deemed satisfactory for further statistical analysis. None of the reported patients had an underlying chronic kidney disease at the time of the CRS and HIPEC. Their characteristics are summarized in Table 1. There were 39 females (67.2%) and 19 males (32.8%). Baseline serum creatinine was 0.81 ± 0.27 (range, 0.29–1.5). Mean BMI was 28.76 ± 7.43 (range, 18–49). Thirty-three patients (56.9%) had a Charlson score≥1. Mean PCI was 17.91 ± 9.12 (range, 0–39). Complete cytoreduction (CC0/1) was achieved in 48 patients (82.7%). The mean length of stay (LOS) was 10.47 ± 4.53 days (range, 5–33). Our CPHQ recorded morbidity in 30 (51.7%) of patients; AKI was the most common and occurred in 12 (20.7%) of patients. Other adverse events included infection/sepsis (12%), pneumonia (8%), cisplatin-related neurotoxicity (7%), *Clostridium difficile* colitis (6%), deep venous thrombosis (DVT) and pulmonary embolism (PE) (6%), and intraabdominal bleeding (1.7%). There was one in-hospital mortality (1.7%) in a patient who developed cerebral edema due to postoperative stroke. There was one 90-day mortality in a patient who developed a GI bleed. Both patients were in the non-AKI group.

**Table 1:** Patients characteristics, N = 58.

Age	58.8 ± 12.4 (21–73)
**Sex**	
Females	39 (67.2%)
Males	19 (32.8%)
Preoperative Creatinine	0.81 ± 0.27 (0.29–1.50)
BMI	28.76 ± 7.43 (18–49)
**Comorbidities**^a^	
Yes	33 (56.9%)
No	25 (43.1%)
PCI	14.91 ± 9.12 (0–39)
CC-0/1	48 (82.7%)
LOS	10.47 ± 4.53 [[5], [6], [7], [8], [9], [10], [11], [12], [13], [14], [15], [16], [17], [18], [19], [20], [21], [22], [23], [24], [25], [26], [27], [28], [29], [30], [31], [32], [33]]
**Clavien-Dindo Complication**	30 (51.7%)
Grade I-II	17 (29.3%)
Grade III-IV	13 (22.4%)
**AKI** (per KDIGO criteria)	12 (20.7%)
0–30 days Mortality	1 (1.7%, cerebral edema)
31–90 days Mortality	1 (1.7%, GI bleed)

Results expressed a mean ± standard deviation (range) or n (percentage). BMI = Body Mass index, PCI = peritoneal carcinomatosis index, CC = completeness of cytoreduction, GI = Gastrointestinal, LOS = length of stay, AKI = acute kidney injury, KDIGO= Kidney Disease: Improving Global Outcomes.

a Comorbidities that affect kidney function in the perioperative period were accounted for, such as the established diagnoses of hypertension, diabetes mellitus, cardiac disease, or systemic autoimmune diseases such as systemic lupus erythematous. Missing variables = none.

Pre-, intra-, and post-operative characteristics of the study population by the occurrence of postoperative AKI are summarized in Table 2, Table 3, Table 4. There was no significant difference in age, sex, BMI, ASA score, and baseline serum creatinine per the occurrence of postoperative AKI. Patients with AKI had a higher rate of pre-existing comorbidities as defined by Charlson score≥1 (10/12, 83.3%) when compared to those without AKI (23/46, 50%; p = 0.037). Intraoperative factors associated with AKI included PCI, operative time, and estimated blood loss (EBL). The use of cisplatin as an intraperitoneal chemotherapy drug vs. other intraperitoneal agents did not influence AKI incidence (p = 0.684), neither did postoperative fluid administration in the initial 24 h. In AKI patients, LOS was 14.2 ± 6.9 days. This was significantly longer than in non-AKI patients (9.5 ± 3.3 days, p = 0.002). Most importantly, patients who suffered from postoperative AKI also endured a significantly higher rate of major surgical morbidities (50.00% vs. 15.21%; p = 0.018). In detail, 6 major morbidities (Clavien III-IV) occurred in the AKI group as follows: 2 patients with pulmonary edema and respiratory failure, 2 pneumonias requiring admission to the ICU, 1 stroke (with subsequent death), and 1 pulmonary embolism (PE); whereas 7 were reported in the non-AKI group: 2 PEs, 1 intraabdominal bleeding, 1 pneumonia, 1 urosepsis, 1 clostridium difficile sepsis, and 1 pulmonary edema and subsequent respiratory failure. Thirty-day readmission rate was not different between the groups. AKI, as defined by an increase in serum creatinine of ≥0.3 mg/dL was noted on postoperative day (POD) 0 in 4 patients and POD1 in 8 patients. Mean time to complete renal recovery occurred on POD 3.12 ± 2.0 (range: 2–8 days).

**Table 2:** Preoperative patient characteristics by the occurrence of postoperative AKI, N = 58.^a^

	AKI Group (N = 12)	Non-AKI Group (N = 46)	P-value
Age	57.8 ± 11.40	54.1 ± 12.7	0.932
Sex			0.963
Female	8	31	
Male	4	15	
BMI (mean ± SD)	28.3 ± 9.3	28.4 ± 7.9	0.307
ASA score (median±SD)	2.8 ± 0.4	2.7 ± 0.6	0.186
Baseline creatinine	0.84 ± 0.27	0.80 ± 0.27	0.876
Charlson score^a^			
0	2 (16.7%)	23 (50%)	**0.037**
≥1	10 (83.3%)	23 (50%)	

Numbers in bold: statistically significant.

AKI = Acute kidney injury, BMI= Body Mass Index, ASA = American Society of Anesthesiology.

a None of the patients had pre-existing chronic kidney disease and their comorbidities were assessed and weighted using the Charlson Comorbidity Index. Missing variables = none.

**Table 3:** Intraoperative patient characteristics by the occurrence of postoperative AKI, N = 58.

	AKI patients (n = 12)	Non-AKI patients (n = 46)	P-value
PCI	20.2 ± 9.1	13.4 ± 8.6	**0.020**
CC-0/1	10 (83.3%)	38 (82.6%)	0.662
EBL (cc)	758.3 ± 633.1	355.4 ± 316.7	**0.001**
Operative time	474.7 ± 136.3	381.8 ± 100.3	**0.011**
Intra-operative fluids (cc)	9346.1 ± 4638.4	7250.4 ± 3021.8	0.196
Intra-operative fluids (cc/hr)	1169.1 ± 362.3	1131.2 ± 352.2	0.743

Numbers in bold: statistically significant.

AKI = acute kidney injury, PCI = peritoneal carcinomatosis index, CC = completeness of cytoreduction, EBL = estimated blood loss. Missing variables = none.

**Table 4:** Postoperative patient characteristics by the occurrence of postoperative AKI, N = 58.

	AKI Group (n = 12)	Non-AKI Group (n = 46)	P-value
Post-operative fluids (cc/24hrs)	6243.6 ± 2600.7	5098.5 ± 2450.6	0.100
All Morbidities	9 (75%)	21 (45%)	0.064
Major Morbidities	6 (50%)	7 (15.21%)	**0.018**
LOS	14.2 ± 6.9	9.5 ± 3.3	**0.002**
30-day readmission	2 (16.7%)	5 (10.8%)	0.626

AKI = acute kidney injury, LOS = length of stay, op = operative. Missing variables = none.

Finally, we performed univariate and multivariate logistic regressions to identify the predictors of the occurrence of AKI, and the predictors of major morbidity occurrence in this patient population. In the former, the univariate model identified Charlson score ≥1, PCI, intraoperative blood loss, intraoperative fluids, and the use of cisplatin as predictors of the occurrence of postoperative AKI. However, all the factors but intraoperative blood loss fell out significant for AKI prediction. Of note, Charlson score and intraoperative cisplatin demonstrated p values of 0.062 and 0.058 in the multivariate model, respectively. Results of the univariate and multivariate regression for predictors of postoperative AKI is presented in Table 5. Similarly, we identified Charlson score ≥1 and postoperative AKI as predictors of major morbidity occurrence following CRS and HIPEC. Age, as a continuous variable, appeared to be a confounder when potential predictors were tested in the multivariate model in our population. Results of this regression is shown in Table 6.

**Table 5:** Univariate and multivariate regression analysis for predictors of AKI occurrence in our CRS and HIPEC population.

	Univariate Analysis		Multivariate Analysis	
	Hazard Ratio [CI]	P	Hazard Ratio [CI]	P
Age	0.983 [0.903–1.070]	0.698	NS	NS
Sex	1.069 [0.111–9.318]	0.954	NS	NS
Charlson score ≥1	2.402 [1.850–5.812]	**0.031**	1.713 [0.921–4.965]	0.062
Baseline creatinine	1.661 [0.020–8.862]	0.450	NS	NS
PCI	1.216 [1.088–1.497]	**0.045**	1.104 [0.908–1.410]	0.140
CC-0/1	1.131 [0.885–1.671]	0.492	NS	NS
Operative time	1.004 [0.985–1.022]	0.696	NS	NS
EBL	2.401 [2.109–2.817]	**0.001**	1.517 [1.462–1.808]	**0.007**
Intraoperative fluids	1.398 [1.091–1.864]	**0.036**	0.920 [0.466–2.185]	0.187
Postoperative fluids	0.910 [0.762–1.824]	0.152	NS	NS
HIPEC agent				
Cisplatin	3.644 [2.115–6.753]	**0.012**	3.017 [0.919–6.741]	0.058
Mitomycin C	1.823 [0.112–3.629]	0.685	NS	NS

Numbers in bold: statistically significant.

AKI = Acute Kidney Injury, CC = Completeness of Cytoreduction, CI = 95% Confidence Interval, CRS = Cytoreductive Surgery, EBL = Estimated Blood Loss, HIPEC = Hyperthermic Intraperitoneal Chemotherapy, PCI = Peritoneal Carcinomatosis Index.

**Table 6:** Univariate and multivariate regression analysis for predictive factors of major morbidity occurrence following CRS and HIPEC in our patients.

	Univariate Analysis		Multivariate Analysis	
	Hazard Ratio [CI]	P	Hazard Ratio [CI]	P
Age	1.852 [1.192–3.017]	**0.040**	1.635 [0.829–3.657]	0.119
Sex	0.917 [0.643–1.381]	0.938	NS	NS
Charlson score ≥1	2.897 [1.936–4.537]	**0.011**	2.013 [1.286–3.168]	**0.044**
PCI	0.739 [0.197–2.528]	0.639	NS	NS
CC-0/1	0.532 [0.099–3.264]	0.939	NS	NS
Postoperative fluids	0.714 [0.163–1.685]	0.761	NS	NS
Postoperative AKI	2.774 [1.653–6.313]	**0.002**	1.855 [1.226–2.431]	**0.030**

Numbers in bold: statistically significant.

AKI = Acute Kidney Injury, CC = Completeness of Cytoreduction, CRS = Cytoreductive Surgery, HIPEC = Hyperthermic Intraperitoneal Chemotherapy, PCI = Peritoneal Carcinomatosis Index.

## 4 Discussion

In the present series, AKI developed frequently following CRS and HIPEC. In fact, it was the most common complication following CRS and HIPEC as recorded by CPHQ.

The RIFLE guidelines, developed in 2004, define AKI as at least a 50% change in serum creatinine relative to a reference value [19]. We evaluated AKI in our cohort of patients using the KDIGO guidelines which have expanded on the RIFLE criteria to include serum creatinine increase as small as 0.3 mg/dL above baseline [20]. When we evaluate larger series of patients undergoing the HIPEC procedure, such terms *nephrotoxicity*, *renal insufficiency*, and *renal failure* are used to describe AKI and are reported in the range 1.3–18.6% [[21], [22], [23], [24], [25]]. In these series, the NCI-CTCAE criteria are often applied and only grade III-V toxicity often reported. This may explain, at least in part, the wide variation of AKI incidence. One study by Hakeam and colleagues specifically reported on the incidence of cisplatin nephrotoxicity post-CRS and HIPEC and found that 3.7% of their patients developed AKI based on the RIFLE consensus criteria [9]. However, they note that serum creatinine was evaluated on POD 3, 7, and 30. This likely led to underreporting of the true incidence of AKI as we have shown that in many patients, AKI is noted within the first postoperative day with full recovery by postoperative day 3. In another study, AKI occurred in 40.4% of ovarian cancer patients undergoing CRS and HIPEC. All those patients received cisplatin [11]. More recently, Cata et al. reported a 21.3% incidence of AKI. This is similar to our results. However, in their cohort of patients, platinum-based infusion was the strongest predictor of postoperative AKI [10]. In our study, multivariate analysis suggested that complex procedures as demonstrated by increased intraoperative blood loss is the strongest predictor of postoperative AKI, with a trend toward significance for pre-existing comorbidities and the use of cisplatin as a HIPEC agent as potential predictors. This could be attributed to the relatively small size of the sample, thus the small number of events, which might have diluted the effect of these factors.

Over the past thirteen years, the adoption of consensus guidelines for AKI allowed for an expanded publication of studies utilizing standardized AKI definitions. It is not surprising that reports on AKI in patients undergoing CRS and HIPEC are lagging. One reason for this is the fact that larger databases in such patients were commenced prior to the adoption of these guidelines. Another reason may be related to the adoption of the American College of Surgeons National Surgical Quality Improvement Program (ACS NSQIP) at many institutions. ACS NSQIP is the largest prospective database that qualifies 30-day risk-adjusted outcomes for patients undergoing major surgical procedures using sampling. Interestingly, it defines postoperative AKI as ‘progressive renal insufficiency’ defined by a postoperative rise in serum creatinine greater than 2 mg/dL. In fact, a study by Dr. Bihorac [26] and her colleagues on 27,841 patients undergoing major surgery reported a 37% incidence of AKI as evaluated by RIFLE and KDIGO consensus criteria and only 7% incidence when evaluated by ACS NSQIP. They concluded that current ACS NSQIP definition underestimates the true incidence of AKI which, in their study, failed to identify most of the patient population who developed AKI and experienced 90-day deaths. Applying ACS NSQIP criteria to our patients similarly underestimates AKI incidence (3 patients, 5.1%) when compared to the KDIGO criteria. Not surprisingly, in another study using 2005–2006 ACS NSQIP database, AKI occurred at a rate of only 1% [27].

Over the past several years and using modern consensus definitions, AKI has been reported with a higher incidence in patients undergoing major non-cardiac surgery. A study analyzing the Veterans Health administration data [28], postoperative AKI occurred in 11.8% of the 161,185 major surgery hospitalizations. In a systemic review, O'Connor et al. [29] reported a 13.4% pooled incidence of postoperative AKI following abdominal surgery. Despite using consensus criteria for their analysis, there was a considerable heterogeneity in the rate of AKI reported ranging from 1.8 to 39%. Our incidence of 20.7% falls within this reported range.

Of the preoperative factors that we studied, only the presence of major comorbidities was associated with a higher rate of AKI. Intraoperative factors that were associated with AKI in our study included PCI, EBL, and operative time, all of which reflect a more extensive and complicated operative course. Thus, indices of complicated operations were logically shown to be important predictors of postoperative AKI [30]. In one recent analysis, sex, hypertension, chronic kidney disease, and ASA physical status classification were reported as independent predictors of AKI [31]. We did not observe that perioperative fluid management influences AKI. Our patients who developed AKI had a higher rate of major morbidity, and a longer length of stay, both of which would typically inflict a higher cost on the healthcare system. The Veterans Health Administration data also showed increased length of stay in patients with AKI after major surgery [1,28]. Many studies have shown associated increase in inpatient mortality with AKI [2,32,33]. Neither our one inpatient mortality nor the one 90-day mortality patients developed AKI. We did not evaluate short-term survival outcome in our patients’ due to the small sample size.

The shortcomings of the current study include its retrospective analysis of prospectively collected data. In addition, our setting is a single medical center which is a community hospital. Thus, the results may not be extrapolated to other patient populations. To our knowledge, however, this is the first report on AKI in CRS and HIPEC using consensus criteria in the setting of not only using cisplatin, but other HIPEC agents as well.

## 5 Conclusion

We conclude that using consensus criteria, AKI incidence is frequent in our patients undergoing CRS and HIPEC at a community center, and correlate with a higher rate of major complications. In addition, ACS-NSQIP definition for AKI significantly underestimates the incidence of this complication. We believe that efforts should focus on prevention and risk management. We are currently implementing clinical pathways of operative quality improvement to reduce major morbidity. However, this work is still in progress, and making a conclusion based on the current data would be premature. Future studies evaluating the true incidence, specific risk factors, and long-term consequences of AKI in HIPEC patients should be conducted. This is important as the data are being submitted to national surgical quality improvement programs.

## Ethical approval

Edward Hospital Institutional Review Board (IRB). This clinical trial is registered under NCT02082886.

## Sources of funding

No funding sources.

## Author contribution

Samer A. Naffouje, MD: data analysis, writing.

Kiara A. Tulla, MD: writing.

Regina Chorley, RN: data collections.

Nancy Armstrong, RN: data collections.

George I. Salti, MD FACS: study design, data collections, data analysis, writing.

## Conflicts of interest

No conflicts of interest.

## Research registration number

None.

## Guarantor

Samer A. Naffouje, MD.

Kiara A. Tulla, MD.

Regina Chorley, RN.

Nancy Armstrong, RN.

George I. Salti, MD FACS.

## Provenance and peer review

Not commissioned, externally peer reviewed.

## Appendix A Supplementary data

AKI paper, the_strocss_statement_checklist AKI paper, the_strocss_statement_checklist
